# Supplementary material for: Evaluation of forearm vascular resistance during orthostatic stress: Velocity is proportional to flow and size doesn’t matter
Source: PLoS One. 2019 Nov 15;14(11):e0224872. doi: 10.1371/journal.pone.0224872 (PMC6857923; doi:10.1371/journal.pone.0224872)
Supplement: Attachment — Submitted filename: Brachial diameter - short report - RESPONSE.docx [file pone.0224872.s001.docx]

Dr. Victoria Claydon

Department of Biomedical Physiology and Kinesiology

Simon Fraser University

8888 University Drive

Burnaby, BC

V5A 1S6

[victoria_claydon@sfu.ca](mailto:victoria_claydon@sfu.ca)

Christopher Torrens

Academic Editor, PLOS ONE

**RE: PONE-D-19-14796 entitled “Evaluation of forearm vascular resistance during orthostatic stress: velocity is proportional to flow and size doesn’t matter”**

Dear Dr. Torrens,

Thank you for your careful consideration of this paper, and for the helpful and constructive reviews of our work. We have carefully considered the reviewer’s comments and have incorporated these suggestions in this revised version of the article. We believe the article is improved as a result and hope it will be acceptable for publication in *PLOS One* in its revised form. We enclose a point-by-point response to the specific comments raised. A copy of the revised manuscript (changes tracked) and in final format (without changes tracked) have been uploaded online.

**JOURNAL REQUIREMENTS**

1. Please ensure that your manuscript meets PLOS ONE's style requirements, including those for file naming.

*We have confirmed compliance with the journal style requirements.*

2. Please provide more information regarding the setting (e.g. locations, relevant dates, periods of recruitment, data collection) and the selection criteria of participants. Please also provide more information regarding participants' baseline characteristics.

*We have expanded the description of the setting in the revised article. We are unsure what baseline characteristics to include. We note the participant’s age, sex, medication use, height, weight, and blood pressure.*

**EDITOR COMMENTS**

This was an interesting paper but it could benefit from some clearer presentation as it was difficult to follow at times. In terms of the test termination, are you saying the only one participants made it through to the -80 mmHg and that the rest terminated from 31 mins (1 min at -40 mmHg) onward? If so it would be nice to see the data of time to termination if only to make things clearer.

*We regret that the participant orthostatic tolerance / test termination times were not clear. The editor is correct in that only one participant completed the entire procedure (to the end of the -60mmHg phase of LBNP). We now clarify this. We could not continue the test to -80mmHg LBNP because this high level of LBNP becomes uncomfortable and becomes technically difficult to sustain. We did not visualise the times to termination but we do now provide the numbers of participants who became presyncopal in each phase.*

**REVIEWER RESPONSES TO QUESTIONS**

1. Is the manuscript technically sound, and do the data support the conclusions?

Reviewer #1: Partly

Reviewer #2: Yes

*We believe the edits made in light of the reviewer’s comments below will increase reviewer one’s confidence in the manuscript.*

2. Has the statistical analysis been performed appropriately and rigorously?

Reviewer #1: Yes

Reviewer #2: I Don't Know

*We are pleased that reviewer one believes the statistical analysis to be appropriate and rigorous.*

3. Have the authors made all data underlying the findings in their manuscript fully available?

Reviewer #1: Yes

Reviewer #2: Yes

*We are pleased the reviewers consider the data to be presented transparently.*

4. Is the manuscript presented in an intelligible fashion and written in standard English?

Reviewer #1: Yes

Reviewer #2: Yes

*We are pleased the English is considered to be of a suitable standard for publication.*

**REVIEWER ONE**

Thank you for your contribution, as this is a well written manuscript with an interesting addition to the study of vascular resistance and orthostatic hypotension. While I find this manuscript interesting and well written, I have questions regarding some of the content.

*We are pleased you consider the work to be well written and interesting. We have considered your questions carefully below.*

The first question regarding the methodology is the measurement of the brachial artery and not an artery closer to the lower extremities. The location of the brachial arteries makes them less likely to react to the changes in flow, as the increase in flow is going to be in the lower extremities during orthostatic hypotension. The logical argument for using the brachial artery is that brachial artery measurement and flow-mediated dilation measurements are well validated. However, in your discussion (line 207-211) you cite a study that combats the validity of FMD measurements in youth and adults. There are hundreds of studies validating the use of FMD for a variety of populations, so I'm lost as to why you would include this study to support your findings. Citing Robergs (1997) brings question as to why you would ever use ultrasound as a tool for measuring vessel diameter changes in response to flow alterations.

*The reviewer’s suggestion that evaluation of a vessel in the lower extremities might be insightful during orthostatic stress is well taken. In fact, we have explored the use of femoral measurements in the past. However, these measurements are not suitable for use in this protocol, because the LBNP chamber covers the lower extremities rendering ultrasound probe placement with high signal quality and a constant angle of insonation prohibitively challenging.*

*We agree with the reviewer regarding the Ostrem (2015) paper, and have removed this section. In the Robergs (1997) paper I think the confusion stems from the differential effects of neural (autonomic) regulation, which seems not to influence brachial arterial diameter but rather the downstream resistance vessels, and local metabolic factors (such as during measures of flow-mediated dilatation with reactive hyperaemia) that do influence brachial vessel diameter.*

In line 126 the authors state the occurrence of vasoconstriction as evidenced by the increase in vascular resistance. With no change in brachial diameter, I recommend the authors be more specific regarding the location of vasoconstriction.

*We now specify the likely site of vasoconstriction. We felt this was better suited to inclusion in the discussion.*

Thank you again for your contribution, and I look forward to seeing the edited version of this manuscript.

**REVIEWER TWO**

Claydon et. al. compared the forearm vascular resistance-- estimated using both brachial arterial flow and velocity.-- in response to supine, tilt, with and without different levels of simulated hypotension (LBNP). Strong correlations were found using both methods. The diameter of the brachial artery remains constant during orthostatic stress and no correlation was found between the basal (supine) diameter and the maximal vascular resistance response. The authors conclude that Doppler-based measurement of brachial velocity can be used to estimate vascular resistance when vascular diameters are unknown. The vascular size does not matter in the context of an individual’s ability to mount a vasoconstrictor response to orthostasis. There are several major concerns:

The current paper seems to validate a basic physiological concept that is already known and accepted--- small arteries and arterioles instead of large vessels (such as the brachial artery with diameter 3-5 mm) are the major components of resistance. Indeed, no diameter changes in the brachial artery were found during pressure compensation. Without significant diameter changes, blood velocity surely correlates with blood flow. The significance and novelty needs to be improved.

*We completely agree with the reviewer. However, we and others using this technique are repeatedly asked to justify the use of brachial velocity as a proxy for flow in publications and presentation, suggesting at least some scepticism in the field around this assumption. This is noted in lines 51-54.*

The description of data is very confusing. For example, it is stated in the Methods that in all cases presyncope occurred at high levels of orthostatic stress, during either -40mmHg or -60mmHg lower body negative pressure, and the maximum vascular resistance response occurred after 31±2.8 minutes of orthostatic stress (corresponding to the first minute of the -40mmHg lower body negative pressure phase). However, only LBNP within 20 mmHg was shown in the Results.

*We are sorry the article was not clear in this regard. We think the confusion arose because the vascular resistance responses were quantified in two ways, using velocity, and flow. We had continuous measures of velocity, but only intermittent measures of flow. We have now tried to clarify how these calculations were derived, and to be more specific in our abbreviations as to which parameter we are using,*

*We did not originally show data from the -40mmHg phase because only 7 participants were able to tolerate the entirety of this phase. Instead we reported data from the supine, tilt and -20mmHg phase. We did state this in the methods. We also now report the numbers of individuals who tolerated each phase. The challenge with the -40mmHg LBNP data is that the sample size for this measure is reduced, and we were not able to obtain measures of brachial diameter at the end of this phase because only 7 participants tolerated the test to this timepoint, and 4 of them were imminently presyncopal at the time. Our focus, therefore was on monitoring our participants and promptly terminating the test to ensure frank syncope was voided, rather than the diameter measurements. We now report the brachial velocity-derived measures at this time in Table 1, but for the reasons stated above, could not include these measures in the other analyses conducted. We have tried to make this clear in the narrative, and in the figures and tables now distinguish “LBNP” as either “LBNP_20_” or “LBNP_40_” throughout.*

In the limitation part, the authors mentioned that all the measures during lower body negative pressure were taken within one minute of the mean orthostatic tolerance of the cohort –close to presyncope for the majority of the cohort. Then why were data of LBNP at 40 mmHg not shown?
How was the maximal FVR% collected? The only information that I found was in the Table legend: “Max FVR (%) reflects analyses over the time point at which the maximum FVR velocity response occurred.” The description of this procedure should be explained in the Method section. In the discussion part (line 228), the authors also claim that the forearm vascular resistance response during maximal orthostatic stress is confirmed by the development of presyncope in all but one of the participants. Do these statements suggest that FVR reached maximal levels upon presyncope? If so why were diameters at Max FVR and presyncope not presented?

*We now present the LBNP_40_ data in Table 1, with the caveats noted above. We now better describe the calculation of the maximum FVR and have clarified our use of acronyms to make it clearer to the reader how these data are determined. Typically, the maximum FVR response precedes presyncope (it wanes or even reverses to dilatation at presyncope). We do not have diameter measurements at the maximum FVR timepoint because we did not have continuous measures of diameter and the maximum FVR response did not necessarily occur conveniently at the predetermined times for the diameter measurements in our protocol, or even at the same time in all individuals.*

*Of note, when reviewing the date in Table 1 as we incorporated the LBNP_40_ data, we spotted an inadvertent data entry error, which we have now corrected. Note that this did not apply to the data represented in the figures, or to the statistical analyses.*

Although the authors pointed out as a limitation, it was still not clear how the high altitude situation may affect tolerance and vascular response to LBNP. The artery size may be unaffected but oxygen carrying capability and tissue oxygenation are likely changed in the participants.

*We agree that the high altitude at which these data were collected has the potential to influence the responses. However, we do not consider it likely that this effect would change over the course of the test, or affect the artery size, and therefore confound the primary question under consideration. Any impact on oxygen carrying capacity or tissue oxygenation is likely to be a constant. The reviewer’s point is well taken, and as the reviewer notes, we did allude to this in the manuscript. We now also mention the associated hypoxia (and hypocapnia) that would accompany high altitude residence. However, we do not believe it invalidates the primary findings of the work.*

The conclusion of “the vascular size does not matter in the context of an individual’s ability to mount a vasoconstrictor response to orthostasis” may be misleading as the vessels used for size measure were not the vessels that constricted due to increase FRV.

*We now clarify this point in the conclusion.*

In Table 1, please clarify FVR calculated by velocity and FVR calculated by flow.

*We now clarified the column headings and legend to Table 1 to make this clear.*

In Figure 2, LBNP (dark close circle) data are missing.

*We apologise for this inadvertent error. The LBNP data are now incorporated.*

Line 167, "normalise" should be "normalize".

*We have corrected this error.*

We thank the reviewers and editorial team for their careful review of this manuscript, and hope it will be acceptable for publication in its revised format.

Best wishes,

Victoria Claydon
